# Effect of atmospheric carbon dioxide levels and nitrate fertilization on glucosinolate biosynthesis in mechanically damaged *Arabidopsis* plants

**DOI:** 10.1186/s12870-016-0752-1

**Published:** 2016-03-22

**Authors:** Jamuna Risal Paudel, Alexandre Amirizian, Sebastian Krosse, Jessica Giddings, Shoieb Akaram Arief Ismail, Jianguo Xia, James B. Gloer, Nicole M. van Dam, Jacqueline C. Bede

**Affiliations:** Department of Plant Science, McGill University, 21,111 Lakeshore, Ste-Anne-de-Bellevue, QC H9X 3V9 Canada; Molecular Interaction Ecology, Radboud University, Heyendaalseweg 135, 6525 AJ Nijmegen, Netherlands; Department of Animal Science, McGill University, 21,111 Lakeshore, Ste-Anne-de-Bellevue, QC H9X 3V9 Canada; Institute of Parasitology, McGill University, 21,111 Lakeshore, Ste-Anne-de-Bellevue, QC H9X 3V9 Canada; Department of Chemistry, University of Iowa, Iowa City, IA 52242 USA; Current Address: German Centre for Integrative Biodiversity Research (iDiv) Halle-Jena-Leipzig, Deutscher Platz 52, D-04103 Leipzig, Germany

**Keywords:** *Arabidopsis thaliana*, Carbon dioxide, Glucosinolate, Nitrate fertilization

## Abstract

**Background:**

Increased atmospheric carbon dioxide (CO_2_) levels predicted to occur before the end of the century will impact plant metabolism. In addition, nitrate availability will affect metabolism and levels of nitrogen-containing defense compounds, such as glucosinolates (GSLs). We compared *Arabidopsis* foliar metabolic profile in plants grown under two CO_2_ regimes (440 *vs* 880 ppm), nitrate fertilization (1 mM *vs* 10 mM) and in response to mechanical damage of rosette leaves.

**Results:**

Constitutive foliar metabolites in nitrate-limited plants show distinct global patterns depending on atmospheric CO_2_ levels; in contrast, plants grown under higher nitrate fertilization under elevated atmospheric CO_2_ conditions have a unique metabolite signature. Nitrate fertilization dampens the jasmonate burst in response to wounding in plants grown at elevated CO_2_ levels. Leaf GSL profile mirrors the jasmonate burst; in particular, indole GSLs increase in response to damage in plants grown at ambient CO_2_ but only in nitrate-limited plants grown under elevated CO_2_ conditions.

**Conclusions:**

This may reflect a reduced capacity of C3 plants grown under enriched CO_2_ and nitrate levels to signal changes in oxidative stress and has implications for future agricultural management practices.

**Electronic supplementary material:**

The online version of this article (doi:10.1186/s12870-016-0752-1) contains supplementary material, which is available to authorized users.

## Background

In response to environmental stresses, plants have evolved an impressive diversity of chemical defenses [1]. In particular, plant specialized metabolites involved in protection against insect herbivores can function as feeding deterrents, antinutritive factors or toxins to protect plant tissues or act as cues to attract natural enemies of plant pests [2]. Synthesizing and maintaining these defense metabolites, such as glucosinolates (GSLs), is costly and plants must efficiently balance the trade-off between growth and defense [3–5]. However the current picture might change as atmospheric carbon dioxide (CO_2_) levels are predicted to rise dramatically, doubling by the end of the century [6]. According to the Scripps Research Institute (Mauna Lao Observatory), monthly mean CO_2_ concentrations have reached 400 ppm and are predicted by the Intergovernmental Panel on Climate Change to reach 880 ppm by 2100 (www.ipcc-data.org/observ/ddc_co2.html). CO_2_ enrichment is known to promote photosynthetic and nitrogen use efficiency, particularly in C3 plants, through an increase in the rate of CO_2_ fixation. Even though, as a consequence, plants are predicted to be more tolerant to nitrogen deficiency, there also may be limitations in readily available nitrogen fertilizers due to increasing production costs. This problem may be exacerbated in countries with limited access to costly farming inputs [7]. Therefore, global changes in atmospheric CO_2_ levels combined with potential limitations of nitrogen fertilizers will alter plant nutrient patterns in agricultural fields.

Understanding how plants adapt to these rapidly changing environmental conditions still remains a challenge [8]. The recent availability of unbiased metabolite profiling to simultaneously measure hundreds of metabolites in plant tissues combined with analysis of underlying metabolic pathways are valuable tools to evaluate metabolic shifts in response to changing environmental conditions to determine the potential impact of nutrient availability on plant defenses [5, 9]. In the first part of this study, an unbiased, exploratory approach was used to gain a “global” view of metabolite profile in vegetative *Arabidopsis thaliana* (L.) Heynh. (Brassicaceae) plants that were grown under conditions of ambient or elevated CO_2_ and fertilized by 1 mM or 10 mM nitrate 440 ppm *vs* 880 ppm. Rosette leaves were wounded and changes in metabolite profile were also monitored.

Thale cress, *Arabidopsis thaliana*, is a fast-growing herbaceous plant [10]. Like most plants in the order Brassicales, the main group of specialized metabolites produced by *A. thaliana* are glucosinolates (GSLs). These nitrogen- and sulfur-rich compounds are constitutively present in plant tissues and their biosynthesis is stimulated by biotic stress and mechanical wounding [11]. The second part of the study focused on GSL biosynthesis in response to CO_2_ and nitrate fertilization.

The basic GSL structure is an *S*-glycosylated thiohydroximate sulfate ester linked to an amino acid derived side chain [12]. *Arabidopsis thaliana* produces ~40 different GSLs that are classified as aliphatic or indole based on the nature of the amino acid precursor [13]. Six R2R3-type MYB transcription factors regulate GSL biosynthesis [14] (Additional file 1: Figure S1). MYB34, MYB51 and MYB122 regulate the expression of genes encoding proteins involved in indole GSL biosynthesis [15]. These three MYB transcription factors show some degree of functional redundancy and tissue-specific expression patterns [16]; MYB34 and MYB122 are predominantly associated with the root tissues, whereas MYB51 is found in leaves [16, 17]. MYB34 positively regulates genes involved in the biosynthesis of Trp and indole-3-acetic acid as well as genes encoding cytochrome P_450_ enzymes CYP79B2/3 and CYP83B1 in the GSL biosynthetic pathway. Overexpression of *AtMYB34* leads to the accumulation of glucobrassicin (3-indolylmethyl GSL, GBC), the most abundant indole GSL in *Arabidopsis* [18]. Overexpression of *AtMYB51* results in the accumulation of indole alkaloids and results in reduced consumption of leaves by caterpillars of the beet armyworm *Spodoptera exigua* [17]. In comparison, MYB122 has a minor but complementary role in indole GSL biosynthesis [15].

In contrast, MYB28, MYB29 and MYB76 positively regulate aliphatic GSL biosynthesis [19, 20]. MYB28 induces the expression of *MAM1/3, CYP79F2* and *ST5b/c* transcripts that encode enzymes in the aliphatic GSL pathway. MYB29 induces the accumulation of short-chain GSLs and may serve as an integrator of signals from MYB26 and MYB76, as it is upregulated by both these transcription factors and has a direct inhibitory effect on MYB28 [21, 22]. Double *myb28/myb29* mutants lacking aliphatic GSLs had significantly reduced resistance to the generalist herbivore *Mamestra brassicae* [23]. MYB76 is believed to play a minor role in the regulation of aliphatic GSL biosynthesis as *Atmyb76* mutants have similar GSL profiles to those of wildtype plants [16]. Sønderby et al. [24] reported that *MYB76* overexpression leads to an increase in long-chained GSLs. MYB28, MYB29 and MYB76 function antagonistically and repress expression of *MYB34, MYB51* and *MYB155* transcripts [16]. How expression of these six key MYB transcription factors that regulate GSL biosynthesis are affected by elevated CO_2_ conditions and nitrate availability is unknown.

The impact of elevated atmospheric CO_2_ on GSL accumulation has been assessed in several Brassicaceae species. Karowe et al. [25] found that a shift in GSL levels was not correlated to the carbon-to-nitrogen ratio of plant tissues as this ratio increased in the tissues of all plant species tested while foliar GSLs increased, decreased or remained unchanged in a stage- and species-specific manner. Other studies have either found no difference in GSL content or slight changes in the concentrations of a few compounds between plants grown under ambient or elevated CO_2_ levels [4, 26–28]. Bidart-Bouzat et al. [4] reported a CO_2_ x herbivory effect in *Arabidopsis* ecotypes Cvi-O and Edi-O; *A. thaliana* plants with lower constitutive defenses accumulate significantly more GSLs after damage by caterpillars of the diamondback moth *Plutella xylostella* under elevated CO_2_ levels. In comparison, GSLs remained unchanged in herbivore-attacked plants grown under ambient CO_2_ levels.

Given that these are nitrogen- and sulfur-rich plant metabolites, the influence of nitrogen availability on GSL biosynthesis has mainly been studied in relation to plant sulfur status in Brassicaceous plants. *Brassica oleracea* var. *capitata* accumulates more GSLs when grown under nitrogen-limited conditions [29]. In comparison, in *Brassica rapa* nitrogen stress led to an overall reduction in GSL accumulation in plants receiving adequate sulfur fertilization [30]. In. *B. oleracea* var *italica* (broccoli) changing nitrogen fertilization rates had a non-linear effect on foliar GSL profiles, suggesting that nitrogen stress favours the synthesis of GBC [31]. In contrast, *B. oleracea* var. *alboglabra* (white cabbage) plants suffering nitrogen stress had higher total GSL levels than those receiving adequate or excess nitrogen fertilization under ambient CO_2_ conditions (350 ppm) [27]. Elevating CO_2_ levels to 800 ppm results in a greater increase in aliphatic and total GSLs in nitrogen-derived plants than in adequately fertilized plants suggesting a significant CO_2_ x nitrogen interaction. Moreover, the increase in carbon-to-nitrogen ratio under CO_2_ enrichment did not lead to depressed GSL levels in bolting stems [27].

Mechanical damage activates hormone-associated signaling pathways that modulate gene expression and lead to the production of specialized metabolites [32, 33]. Key wound-activated octadecanoid signaling molecules are 12-*oxo*-phytodienoic acid (OPDA), jasmonic acid (JA) and the biologically active form of JA, 7-jasmonoyl-L-isoleucine (JA-Ile) [34]; cellular increases in these hormones lead to wound-induced plant responses [35, 36]. Changes in abscisic acid (ABA) levels are also often observed in response to wounding, possibly as a response to water losses at the site of damage [37]. In contrast, constitutive salicylic acid (SA) levels increase as part of the hypersensitive response to pathogens leading to systemic acquired resistance [38, 39]. Between all these hormones, there can be cross-talk between signaling pathways that modify the final metabolic response [40] (Additional file 1: Figure S1).

Mechanical wounding activates the expression of *MYB28, MYB29, MYB51, MYB76* and *CYP79B2/3* [16, 24, 41, 42]. However, there is limited information on the effects of wounding on GSL levels. Higher levels of indole GSLs, GBC and 4-hydroxy-3-indolylmethyl-GSL (4HO3IM), were found after wounding or feeding by crucifer specialist flea beetle *Phyllotreta cruciferae* damage of *B. napus* cv. ‘Tobin’ and *B. juncea* cv. ‘Cutlass’ compared to *B. napus* cv. ‘Westar’ where only GBC levels increased [43]. In contrast, wounding did not affect GSL levels in *Sinapis alba* cv. ‘Ochre’ cotyledons, but this species has higher constitutive GSL levels compared to other plant species. In *A. thaliana*, foliar indole GSL levels (i.e. 4-hydroxy-3-indolylmethyl GSL) increased 24 h after damage by ribbed forceps [41]. In comparison, levels of aliphatic GSLs (i.e. 8-methylthiooctyl GSL and 8-sulphinyloctyl GSL) and indole GSLs (i.e. GBC and *N*-methoxy-3-GBC) increased after methyl jasmonate treatment.

Bidart-Bouzat and Imeh-Nathaniel [44] stress the need to study CO_2_-dependent changes in stress-induced foliar defense metabolites profiles as they could provide valuable predictions on future plant-herbivore interaction patterns. As nitrogen supply is known to affect plant responses to CO_2_ enrichment [45], these two factors need to be studied simultaneously to accurately predict the outcome of increasing atmospheric CO_2_ changes on plant defense mechanisms. The aim of this project is to evaluate the combined effects of CO_2_ enrichment and nitrate levels on metabolite levels in mechanically damaged *A. thaliana* leaves. A non-targeted approach using liquid chromatography-quadrapole time-of-flight mass spectrometry (LC-Q-TOF-MS) was used to explore overall patterns of foliar metabolites occurring in response to these environmental stresses [46]. This was followed by a focused study on the foliar phytohormone and GSL transcription factor expression and levels. In response to plant damage (i.e. wounding), a shift from aliphatic to indole GSLs is often observed [41, 47, 48]. Therefore, we measured the expression of key MYB transcription factors involved in the regulation of GSL biosynthesis. In *Arabidopsis*, mechanical damage induces the expression of *MYB28* and *MYB29*, responsible for the regulation of genes encoding enzymes in the aliphatic GSL pathway, as well as *MYB 51*, responsible for the regulation of genes encoding enzymes in the indole GSL pathway [16, 24, 41, 42]. These two pathways are antagonistic; MYB factors in the indole pathway are believed to downregulate the aliphatic pathway and vice versa [23].

## Methods

### Plant growth conditions

*Arabidopsis thaliana* (Col-0, obtained from The Arabidopsis Information Resource (TAIR)) seeds were cold stratified at 4 °C for two days to obtain a constant germination rate [49]. After sowing in 16 cm pots containing Fafard PV20 agromix, pots were transferred to one of two growth cabinets (16:8 h light:dark, 250 μE m^−2^ s^−1^, 23 °C) under ambient (440 ppm) or elevated (880 ppm) CO_2_. After two weeks, seedlings were transferred to pots and randomly assigned to one of the two fertilization groups; the first set was subjected to nitrogen stress (1 mM nitrate) and the second group was given sufficient nitrate (10 mM nitrate). To make up these fertilizers, concentrations of all other components were the same with the exception of Cl^−^; the difference in Cl^−^ concentration is considered insignificant as it is at a supra-optimal level and below potentially toxic levels [50, 51]; Cl^−^ was approximately 9 mM higher in the nitrogen-stressed plants. Plants were fertilized every two days with watering.

### Wound treatment and sample collection

At approximately 6 weeks (stage 3.9 [52]), half of the plants for each treatment were randomly selected to be mechanically damaged; approximately 20 % of each rosette leaf in the mechanically wounded treatment was removed using a hole punch. To minimize volatile signalling between different groups of plants, a plexiglass panel was placed between wounded and control plants. After 24 h, the entire rosette was harvested and flash frozen in liquid nitrogen and stored at −80 °C until subsequent analysis. For hormone, gene expression, GSL and untargeted metabolomic analyses, two biological replicates were taken and the experiment was temporally repeated (total n = 4 independent biological replicates for each analysis).

### Untargeted metabolite extraction and mass spectrometry

Metabolite extraction and analysis was conducted as described by de Vos et al. [46]. Lyophilized leaf samples were finely ground using a TissueLyzer (15 Hz s^−1^) and metabolites extracted in 75 % aqueous methanol (MeOH) acidified with 0.125 % formic acid (v/v). Following vigorous vortexing (10 s) and sonication (40 kHz, 20 min in a water bath maintained at 20 °C), samples were centrifuged at 20,000 g for 10 min and the supernatants transferred to clean tubes. Supernatants were then filtered through 0.2 μm PTFE syringe filters and transferred to HPLC vials.

Metabolite separation and identification was performed by ultra-performance liquid chromatography (UPLC) interfaced with a quadrupole time-of-flight hybrid mass spectrometer (Q-TOF-MS; Waters) at the High Resolution Mass Spectrometry Facility at the University of Iowa. Randomized samples were separated on a C_18_ (Waters Acquity BEH, 2.1 x 100 mm, 1.7 μm) column using a gradient solvent at a flow rate of 0.2 mL/min; the mobile phase was increased from 5 % acetonitrile (ACN) with 0.1 % formic acid to 75 % ACN with 0.1 % formic acid over 20 min, 75 % ACN with 0.1 formic acid was maintained for 5 min and then ACN levels were lowered to initial conditions over 1 min and re-equilibrated for 4 min. Column temperature was maintained at 40 °C. For MS detection, negative mode electrospray ionization (ESI) was used and data were collected in the centroid mode following the procedure described by de Vos et al. [46]. Full scan mass spectra for the ions in the mass range of 100–1500 Da were collected every 900 ms with an interscan delay of 100 ms.

### Liquid chromatography-mass spectrometry (LC-MS) processing and metabolite identification

Data pre-processing and alignment was performed with MzMine program (version 2.10) [53]. Briefly, raw data from the Waters Q-TOF-MS were converted to Net CDF format. In MzMine, data were filtered using the Savitzky-Gravity filter, then base-corrected and peaks were detected in the centroid mode. The chromatogram was built using a 0.05 m/z tolerance and deconvoluted using the algorithm “Local Minimum Search”. Peaks were aligned using the “Join Aligner” algorithm allowing 100 ppm m/z tolerance and followed by gap filling (Additional file 2: Table S1). Changes in metabolite levels (peak areas) were analyzed using MetaboAnalyst 3.0 (www.metaboanalyst.ca; [54–56]). Within each treatment (i.e. stress), data were analyzed as 2-factor independent samples. Data were filtered using an interquantile range (IQR) and log-transformed and auto-scaled (mean-centered and divided by the SD of each variable) to normalize the data. An overview of the data was then observed using Principal Component Analysis (PCA) and Heatmaps to understand global patterns.

### Phytohormone extraction and analysis

Lyophilized and finely ground foliar tissues were sent to the Proteomics and Mass Spectrometry Facility at the Danforth Plant Science Center (Missouri, USA) for the analysis of phytohormone (JA, JA-Ile, OPDA, SA, and ABA) levels by liquid chromatography-tandem mass spectrometry. Plant samples were spiked with deuterium-labelled internal standards of SA (D^5^-SA), ABA (D^6^-ABA), and JA (D^2^-JA) and extracted in ice cold MeOH:ACN (1:1, v/v). Following centrifugation (16,000 g), the supernatants were collected and pellet extraction was repeated. The pooled supernatants were evaporated using a speed-vac and the resulting pellet redissolved in 30 % MeOH.

Phytohormone analysis was performed using a C_18_ column (Onyx, 4.6 mm × 100 mm, Phenomenex). Separation was achieved using a mobile gradient of 40 % solvent A (0.1 % acetic acid in HPLC-grade water, v/v) to 100 % solvent B (0.1 % acetic acid in 90 % ACN, v/v) over 5 min at a flow rate of 1 mL min^−1^. A 4000-QTRAP (AB Sciex) was used to obtain the mass spectra using parameters set as follows: ESI in the negative mode (TurbolonSpray), capillary voltage −4500, nebulizer gas (N_2_) 50 arbitrary units (a.u.), heater gas 50 a.u., curtain gas 25 a.u., collision activation dissociation high, temperature 550 °C. Compounds were detected using multiple reaction monitoring transitions that were optimized for each phytohormone and deuterium-labelled standards [57]. Concentrations were determined from standard curves of known compounds.

### Glucosinolate (GSL) extraction and analysis

GSLs were extracted and analyzed following van Dam et al. [58]. Briefly, 50 mg of lyophilized leaf material was finely ground using a TissueLyser. Following incubation at 90 °C for 6 min to inactivate plant myrosinases, samples were ultra-sonicated for 15 min in 70 % MeOH. After centrifugation at 2975 g for 10 min, the supernatant was transferred to a clean tube and the pellet was re-extracted. Supernatants were pooled and cleaned up using a diethylaminoentyl Sephadex A-25 ion exchange column preconditioned with sterile MilliQ water. After washing with 70 % MeOH (2 × 1 mL), MilliQ water (2 × 1 mL) and 20 mM sodium acetate buffer, pH 5.5 (1 × 1 mL), GSLs were treated with 10 U of arylsulfatase and incubated at RT for 12 h. The desulfated GSLs were eluted in sterile MilliQ water (2 × 0.75 mL) and the eluants were lyophilized.

GSL extracts were separated by high performance liquid chromatography (DIONEX summit HPLC). Compounds were separated on a reverse-phase C18 column (Alltima C18, 150 × 4.6 mm, 3 μm, Alltech) using a mobile gradient from 2 % ACN to 35 % ACN in 30 min at a flow rate of 0.75 mL min^−1^. Compounds were detected by a photodiode array detector (DAD) at 229 nm (EC, 1990). GSLs were identified based on retention time, UV spectra and mass spectra. Reference standards of GSLs (glucoiberin (3-methylsulfenylpropyl GSL), glucoerucin (4-methylthiobutyl GSL), progoitrin (2-hydroxy-3-butenyl GSL), sinigrin (2-propenyl GSL), gluconapin (3-butenyl GSL), glucobrassicanapin (4-pentenyl GSL), glucobrassicin (indol-3-ylmethyl GSL), sinalbin (4-hydoxybenzyl GSL), glucotropaeolin (benzyl GSL), and gluconasturtiin (2-phenylethyl GSL); Phytoplan, Heidelberg, Germany) were employed as standards in the HPLC analysis. Correction factors were used to calculate GSL concentrations from an external sinigrin standard curve [59–61].

### Gene expression analysis

Total RNA was extracted from leaf tissue samples using an RNeasy Plant Mini kit (Qiagen) according to the manufacturer’s instructions. RNA quality and concentration were determined spectrophotometrically (Infinite M200 Pro plate reader, Tecan). The absence of DNA contamination was verified by polymerase chain reaction (PCR) using primers designed against an intronic region of Ethylene-Insensitive-Like2 (*EIL2*) (5′-CAGATTCTATGGATATGTATAACAACAA-3′ and 5′-GTAAAGAGCAGCGAGCCATAAA G-3′) [62]. PCR amplicons were separated on a 1 % gel. Genomic DNA was included as a positive control.

The relative transcript expression of MYB transcription factors involved in GSL biosynthesis (*MYB28*, *MYB29*, *MYB76*, *MYB34*, *MYB51* and *MYB122*) was measured by quantitative real time-PCR (qRT-PCR, MX3000p thermo-cycler, Stratagene) using absolute blue SYBR green with low ROX (Fisher Scientific). The qRT-PCR reaction contained 1 x SYBR green, cDNA (1/10 dilution) and 80 nM of gene-specific forward and reverse primers (Additional file 3: Table S2). The thermal cycling program was: 95 °C for 10 min followed by 40 cycles of 95 °C for 15 s, 58-60 °C for 30 s (temperature dependent on the primer pair, Additional file 3: Table 2) and 72 °C for 30 s. The presence of a single amplicon was confirmed by a sharp dissociation curve. Samples were analyzed in duplicate and two technical plates were performed. Non-template controls were included on every plate.

The relative expression of the target genes was calculated as a ratio (R0_GOI_/R0_REF_) to the geometric mean of two reference genes (*AtACT2/7* and *AtUnk*) where the initial template concentration, R0, was calculated using the formula R0 = 1/(1 + E)^Ct^ where E is the average efficiency of gene in the exponential phase and Ct is the threshold cycle [63].

### Statistical analysis

For hormone, gene expression and GSL analyses, statistically significant differences (p ≤ 0.05) were identified by conducting a 3-factorial analysis of variance (ANOVA) using SPSS (vers. 20). When significant interactions were detected, differences were further teased apart by the appropriate 2-way ANOVA (Additional file 4: Table S3). The effect of mechanical damage was determined by comparing constitutive *vs* wounded levels using a Student’s *t*-test.

## Results and discussion

### Global changes in *Arabidopsis* foliar metabolite profile

**Fig. 1:**
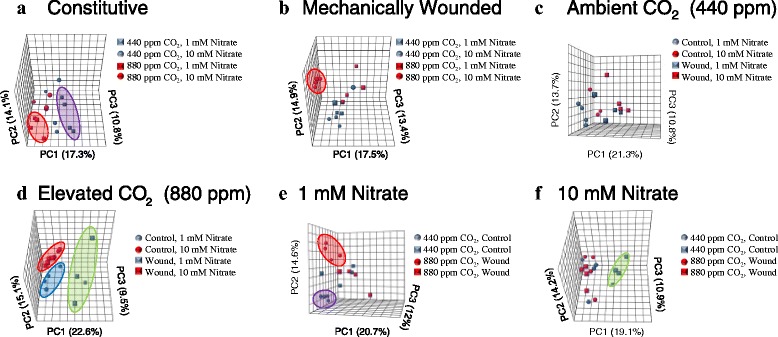
Principal component analysis (PCA) of foliar metabolite profiles of *Arabidopsis* grown at different levels of CO_2_, nitrate fertilization and wounding stress. Plants were grown under two different atmospheric CO_2_ levels (ambient (440 ppm; LC) or elevated (880 ppm; HC)) and fertilized with either 1 mM or 10 mM nitrate and either not treated (control) or mechanically damaged (wound). The average of 4 independent samples were compared by 2-way analysis of variance. **a** Constitutive foliar metabolic profile. The purple shaded area denotes constitutive metabolites extracted from plants fertilized by 1 mM nitrate and grown at ambient CO_2_ compared to the red shaded area denotes constitutive metabolites extracted from plants grown at elevated CO_2_ levels. **b** Wound-induced metabolite profile. The red shaded area denotes metabolites extracted plants fertilized with 10 mM nitrate and grown under elevated atmospheric CO_2_. **c** Metabolite profile of plants grown at ambient CO_2_ levels (440 ppm). The blue shaded area denotes constitutive metabolites extracted from plants fertilized by 1 mM nitrate. **d** Metabolite profile of plants grown at elevated CO_2_ levels (880 ppm). Blue shaded area denotes constitutive metabolites compared to the green shaded area that denotes wound-induced metabolites extracted from plants fertilized with 1 mM nitrate. The red shaded area denotes constitutive and wound-induced metabolites extracted from plants fertilized with 10 mM nitrate. **e** Metabolite profile of plants fertilized with 1 mM nitrate. The purple shaded area denotes constitutive metabolites extracted from plants grown at ambient CO_2_ levels compared to the red shaded are that denotes constitutive metabolites extracted from plants grown at elevated atmospheric CO_2_. **f** Metabolite profile of plants fertilized with 10 mM nitrate. Green circle denotes wound-induced metabolites extracted from plants grown at ambient CO_2_ levels

In *Arabidopsis*, the constitutive foliar metabolite profile of plants grown at lower nitrate fertilization levels (1 mM) strongly reflects atmospheric CO_2_ levels (Fig. 1a, c, e, Additional file 5: Figure S2). PCA analysis segregates constitutive metabolites in plants grown under ambient or elevated CO_2_ levels in nitrate-limited plants. This stark differentiation between metabolite profiles was absent when plants were fertilized by 10 mM nitrate. In response to mechanical wounding, induced metabolic profiles are affected by atmospheric CO_2_ levels and nitrate fertilization (Fig. 1b, f, Additional file 5: Figure S2). At ambient CO_2_ levels, wound-induced metabolite profiles are similar, regardless of nitrate fertilization (Fig. 1c, Additional file 5: Figure S2). This same general pattern is seen in plants grown at elevated CO_2_ under nitrate-limited conditions. A strikingly different wound-induced metabolite profile is seen in plants grown at elevated CO_2_ and high nitrate fertilization (10 mM) (Fig. 1b, f, Additional file 5: Figure S2). At elevated CO_2_ levels, the strong difference in induced metabolite profile observed in plants fertilized with 1 mM nitrate is not as pronounced in plants fertilized with the higher nitrate level (Fig. 1d, Additional file 5: Figure S2).

### Under elevated CO_2_ conditions, the jasmonate burst is limited by nitrate excess

**Fig. 2:**
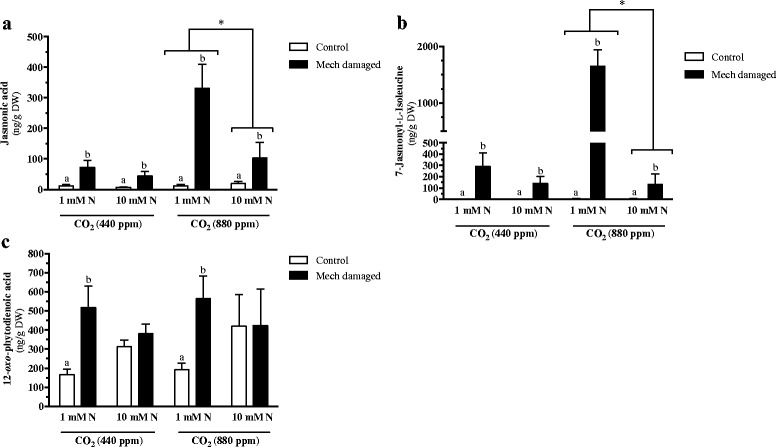
Foliar phytohormones in *Arabidopsis* grown at different levels of CO_2_, nitrate fertilization and wounding stress. Plants were grown under ambient (440 ppm) or elevated (880 ppm) carbon dioxide (CO_2_) levels and fertilized with either 1 mM or 10 mM nitrate (1 mM N or 10 mM N). At 6 weeks, *Arabidopsis* rosette leaves were mechanically damaged (mech. damaged, black bars) or untouched (control, white bars) and phytohormone levels analyzed. **a** Jasmonic acid (JA) **b** 7-Jasmonoyl-L-isoleucine (JA-Ile) **c** 12-*oxo*-phytodienoic acid (OPDA). Statistical differences were determined by 3-factor analysis of variance (Additional file 4: Table 3). When interactions were significant, data were separated to show treatment effects. Significant differences in response to wounding are denoted by alphabetical letters (*p* ≤ 0.05). An asterisk indicates significant differences between grouped variables

Under ambient and elevated CO_2_ levels, JA and JA-Ile levels, representing a strong jasmonate burst, increase in response to wounding (Fig. 2a, b, Additional file 4: Table S3). At elevated CO_2_ levels, an enhanced JA burst is observed in wounded plants that are subject to the lower nitrogen fertilization regime (1 mM nitrate) (Fig. 2a); this is over three times the level of JA induced in plants fertilized by 10 mM nitrate. A similar but more striking increase is observed in the biologically active form of JA, JA-Ile, where, under conditions of elevated CO_2_, a 12-fold difference in induced JA-Ile level is observed between plants fertilized with 1 mM or 10 mM nitrate (Fig. 2b). Therefore, nitrate fertilization dampens the wound-induced jasmonate response in plants grown under elevated CO_2_. Sun et al. [64] also observed that *Arabidopsis* plants grown at elevated atmospheric CO_2_ levels showed a decline in jasmonate-dependent defenses in response to attack by the peach aphid, *Myzus persicae*. Increases in the level of the signaling molecule and biosynthetic precursor to JA, OPDA, is only observed in mechanically damaged plants fertilized with 1 mM nitrate, regardless of the CO_2_ environment (Fig. 2c).

### Glucosinolate biosynthesis and levels

**Fig. 3:**
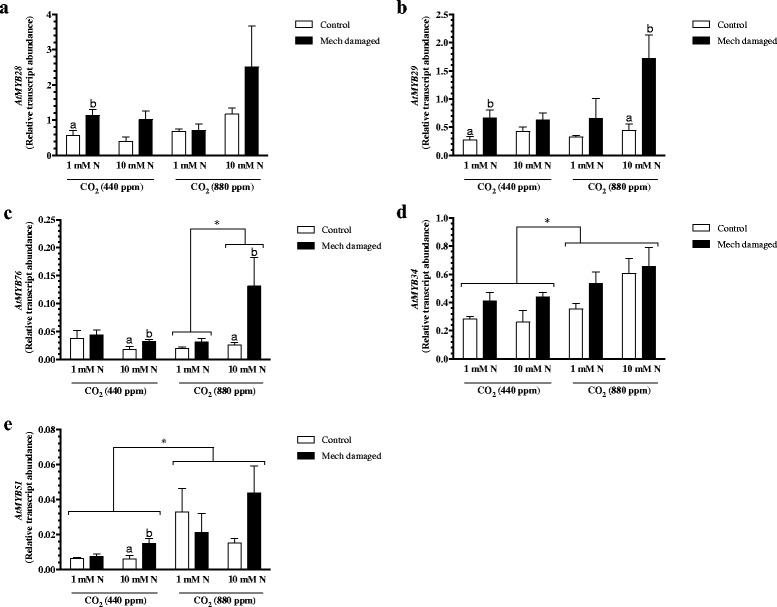
Foliar *MYB* transcription factor gene expression in *Arabidopsis* grown at different levels of CO_2_, nitrate fertilization and wounding stress. *Arabidopsis* plants were grown under ambient (440 ppm) or elevated (880 ppm) carbon dioxide (CO_2_) levels and fertilized with either 1 mM or 10 mM nitrate (1 mM N or 10 mM N). At 6 weeks, rosette leaves were mechanically damaged (mech. damaged, black bars) or untouched (control, white bars). Expression of transcription factors that regulate glucosinolate biosynthesis **a**) *AtMYB28* **b**) *AtMYB29* **c**) *AtMYB76* **d**) *AtMYB34* and **e**) *AtMYB51*. Statistical differences were determined by 3-factor analysis of variance (Additional file 4: Table 3). When interactions were significant (*p* ≤ 0.05), data were separated to show treatment effects. In CO_2_ levels and nitrate fertilization treatments, only significant changes in gene expression are highlighted. Significant differences in response to treatments are denoted by alphabetical letters

The key *Arabidopsis* defensive compounds, GSLs, are nitrogen-rich compounds, therefore, CO_2_ enrichment and nitrogen-limitation may influence their biosynthesis and levels and, ultimately, influence plant resistance to herbivory. Focusing on MYB TFs that regulate aliphatic GSL biosynthesis, *AtMYB28, AtMYB29* and *AtMYB76*, 3-way ANOVA analyses detected an induction in response to wounding but this was often not identified by Student’s *t*-test (Fig. 3a-c, Additional file 4: Table S3). Expression of *AtMYB76* reflects nitrate and atmospheric CO_2_ conditions (Fig. 3c); *Arabidopsis* grown under higher atmospheric CO_2_ levels have increased *AtMYB76* transcript levels when plants are fertilized at a higher nitrate rate. Overall, these results suggest that in response to wounding of plants grown at elevated CO_2_, one might expect an increase in aliphatic GSL levels.

**Fig. 4:**
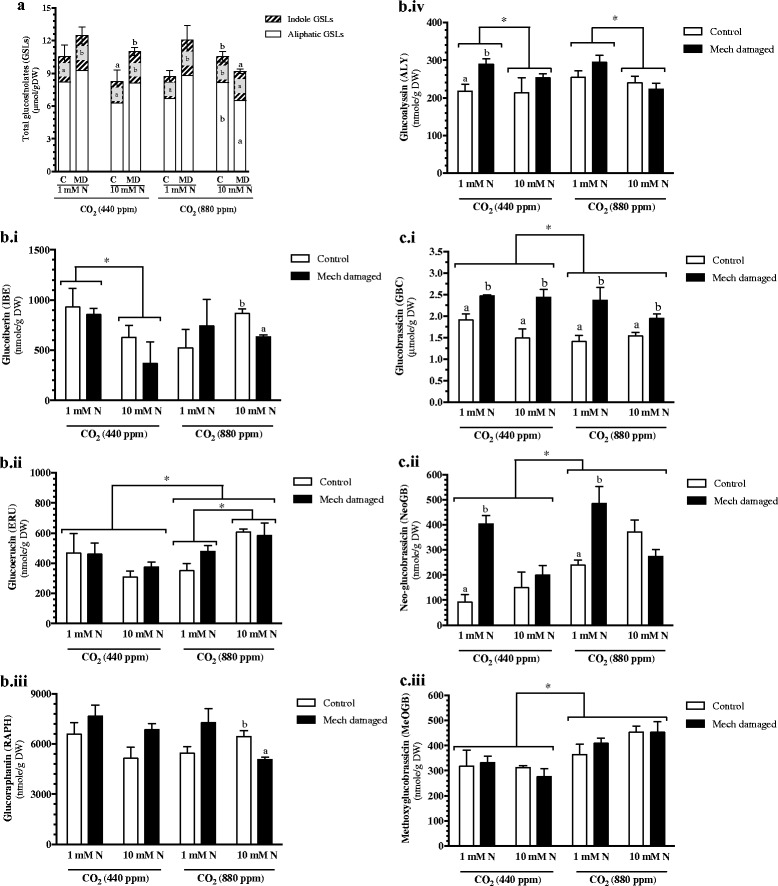
Foliar glucosinolate levels in *Arabidopsis* grown at different levels of CO_2_, nitrate fertilization and wounding stress. Plants were grown under ambient (440 ppm) or elevated (880 ppm) carbon dioxide (CO_2_) levels and fertilized with either 1 mM or 10 mM nitrate (1 mM N or 10 mM N). At 6 weeks, rosette leaves were mechanically damaged (Mech damaged) or untouched (control, C). **a** Total glucosinolates, white bars represent aliphatic GSLs and hatched bars represent indole GSLs. Significant differences (*p* ≤ 0.05) in response to wounding of aliphatic GSLs are denoted inside the white bar, of indole GSLs are denoted inside the hatched bar and total GSLs are denoted on top of the bar by alphabetical letters. **b** Aliphatic glucosinolates *Bi*) Glucoiberin (IBE; 3-methylsulfinylpropyl GSL), *Bii*) Glucoerucin (ERU; 4-methylthiobutyl GSL), *Biii*) Glucoraphanin (RAPH; 4-methylsulfinylbutyl GSL) and Biv) **d**) Glucoalyssin (ALY; 5-methylsulfinylpentyl GSL). **c** Indole glucosinolates *Ci*) Glucobrassicin (GBC), *Cii*) Neo-glucobrassicin (NeoGB) and *Ciii*) Methoxyglucobrassicin (MeOGB). Statistical differences were determined by 3-factor analysis of variance (Additional file 4: Table S3). When interactions were significant, data were separated to show treatment effects. Significant differences in response to wounding are denoted by alphabetical letters (*p* ≤ 0.05). An asterisk indicates significant differences between grouped variables

Overall, a significant increase in total foliar levels of aliphatic GSLs was not observed in response to these treatments (Fig. 4a). In fact, a decrease in aliphatic glucosinolates is observed in wounded plants grown under elevated CO_2_ conditions and high fertilization. This was unexpected given the expression of the MYB transcription factors known to regulate aliphatic GSL biosynthesis and points to the possible multiple levels of regulation of aliphatic GSL biosynthesis as proposed by Burows et al. [65]. A closer inspection of specific aliphatic GSLs shows that levels of the aliphatic 3C class 3-methylsulfinylpropyl GSL glucoiberin (IBE) and 4C class 4-methylsulfinylbutyl GSL glucoraphanin (RAPH) are lower in wounded, nitrate-fertilized plants grown at elevated CO_2_ levels (Fig. 4Bi, Biii).

Aliphatic glucosinolate levels show strong nitrate fertilization x atmospheric CO_2_ interaction. In plants grown at ambient CO_2_, levels of the 4C class 4-methylthiobutyl GSL glucoerucin (ERU) are lower than in plants grown at higher CO_2_ levels (Fig. 4Bii). Unexpectedly, for IBE and the 5C class 5-methylsulfinylpentyl GSL glucoalyssin (ALY), higher levels of these compounds were observed under lower nitrate fertilization rates in plants grown at ambient CO_2_ conditions (Fig. 4Bi, Biv). In contrast, in *Arabidopsis* plants grown under enriched CO_2_ conditions, nitrate fertilization has a positive effect on ERU levels and negatively effects ALY levels (Fig. 4Bi, Biv).

AtMYB34, AtMYB51 and AtMYB 122 regulate indole GSL biosynthesis [15]; AtMYB51 plays the predominant role in regulating foliar indole GSL biosynthesis in *Arabidopsis* leaves [15]. Expression of *AtMYB34* and *AtMYB51* is higher in plants grown at elevated CO_2_ levels (Fig. 3d, e). In comparison with previous reports [17], *AtMYB51* is not strongly induced in response to mechanical damage (Fig. 3a); wound-induced AtMYB51 expression was only observed in plants fertilized with 10 mM nitrate grown at ambient CO_2_ levels. Expression of both *AtMYB34* and *AtMYB51* was higher in plants grown under elevated CO_2_ conditions (Fig. 3d, e). *AtMYB122* expression levels were below the detection limits used in this experiment.

Unlike aliphatic GSLs, atmospheric CO_2_ rather than a CO_2_ x nitrate interaction appears to play a greater role in influencing indole GSL levels. Increased foliar levels of 4-methoxy-3-indolylmethyl GSL (4-methoxyglucobrassicin; 4MeOGB) and 1-methoxy-3-indolylmethyl GSL (neo-glucobrassicin; NeoGB) levels and lower levels of their biosynthetic precursor 3-indolylmethyl GSL (glucobrassicin; GBC) are observed in *Arabidopsis* grown under elevated CO_2_ conditions (Fig. 4Ci-iii); this suggests increased flux toward methoxy-indolyl GSLs under enriched CO_2_ levels. Total foliar indole GSL levels increase in response to wounding under all environmental conditions except in plants grown under nitrogen- and CO_2_-fertilization (Fig. 4). In particular, GBC levels are strongly induced in response to mechanical damage (Fig. 4Ci). NeoGB levels are induced only when plants are grown under nitrogen-stressed conditions (Fig. 4Cii).

## Conclusions

Atmospheric CO_2_ levels and nitrate fertilization play an important role in shaping the constitutive and wound-induced metabolic profile in *Arabidopsis* leaves. Constitutive metabolic profiles reflect atmospheric CO_2_ levels, particularly under nitrate-limited conditions (Fig. 1a, Additional file 5: Figure S2). In contrast, at elevated CO_2_, wound-inducible metabolites show a distinct profile in response to high nitrate fertilization (Fig. 1b, Additional file 5: Figure S2). In fact, the enhanced jasmonate burst observed in wounded *Arabidopsis* grown under an enriched CO_2_ atmosphere is suppressed in plants fertilized with the higher rate of nitrate (Fig. 2a, b). This is also reflected in GSL levels (Fig. 4a). Wound-induced levels of the indole GSL GBC and NeoGB are observed (Fig 4Ci, Cii), although the increased levels of NeoGB are only seen in nitrate-limited plants. As well, a wound-related reduction in the aliphatic GSLs IBE and RAPH is observed in plants grown in enriched CO_2_ and nitrate conditions (Fig. 4Bi, Biii).

One possible explanation for these results is that under elevated atmospheric CO_2_ levels, as photorespiration decreases in C3 plants, cytosolic malate, an important source of reducing power (NADPH) for nitrate assimilation, also decreases [66]. If the readily available NADPH is used for nitrate assimilation, then this may affect the cellular redox balance and the plants’ ability to respond to stresses, such as mechanical damage, may be impaired [67]. We are presently further investigating this possibility. Results from this study suggest that changing atmospheric conditions and nitrate fertilization may affect the plant’s ability to identify and cope with oxidative stress, such as in response to insect damage [68], and, therefore, has important implications for future agricultural management practices for C3 crops.

## Availability of data and materials

Untargeted metabolomic data is available through Additional file 2: Table S1. In addition, metabolomic and glucosinolate data is deposited at http://idata.idiv.de/DDM/Data/ShowData/199 and http://idata.idiv.de/DDM/Data/ShowData/204 sites, respectively. 

## Additional files

Additional file 1: Figure S1. MYB transcription factor regulation of glucosinolate biosynthesis in *Arabidopsis*. Wounding and wound-related stress hormones, such as jasmonic acid or methyl jasmonate, regulate MYB transcription factors that regulate glucosinolate biosynthesis. AtMYB28, AtMYB29 and AtMYB76 regulate expression of genes that encode enzymes in aliphatic GSL biosynthesis whereas AtMYB 34, AtMYB51 and AtMYB122 regulate expression of genes that encode enzymes in indole GSL biosynthesis. Abbreviations: abscisic acid: ABA, glucosinolate: GSL, jasmonic acid: JA, salicylic acid: SA. (PDF 7 kb) Additional file 2: Table S1. Foliar metabolites of *Arabidopsis* plants grown at different CO_2_ levels, nitrate fertilization and wounding stress detected by HPLC-Q-TOF-MS. (XLS 1039 kb) Additional file 3: Table S2. Primers used for quantitative real time-polymerase chain reaction (qRT-PCR) (DOC 40 kb) Additional file 4: Table S3. Statistical analysis of phytohormone, *AtAMB* gene expression and glucosinolate levels. (DOC 94 kb) Additional file 5: Figure S2. Heat map of foliar metabolite profiles of *Arabidopsis* grown at different CO_2_levels, nitrate fertilization and wounding stress. Plants were grown under two different atmospheric CO_2_ levels (ambient (440 ppm; LC) or elevated (880 ppm; HC)) and fertilized with either 1 mM (LN) or 10 mM (HN) nitrate and either not treated (control) or mechanically damaged (wound). The average of 4 independent samples were compared by 2-way analysis of variance. Metabolites are identified by retention time/mass over charge ratio (RT/m/z). A) Constitutive foliar metabolic profile. Factors; 1 mM nitrate (blue), 10 mM nitrate (pink), 440 ppm CO_2_ (green) and 880 ppm CO_2_ (purple). B) Wound-induced metabolite profile. Factors; 1 mM nitrate (blue), 10 mM nitrate (pink), 440 ppm CO_2_ (green) and 880 ppm CO_2_ (purple). C) Metabolite profile of plants grown at ambient CO_2_ levels (440 ppm). Factors; constitutive (pink), mechanically damaged (blue), 1 mM nitrate (green) and 10 mM nitrate (purple). D) Metabolite profile of plants grown at elevated CO_2_ levels (880 ppm). Factors; constitutive (pink), mechanically damaged (blue), 1 mM nitrate (green) and 10 mM nitrate (purple). E) Metabolite profile of plants fertilized with 1 mM nitrate. Factors; control (pink), mechanically damaged (blue),440 ppm CO_2_ (green) and 880 ppm CO_2_ (purple). F) Metabolite profile of plants fertilized with 10 mM nitrate. Factors; control (pink), mechanically damaged (blue),440 ppm CO_2_ (green) and 880 ppm CO_2_ (purple). (PDF 204 kb)

## Abbreviations

4HO3IM: 4-hydroxy-3-indolylmethyl-GSL
4MeOGB: 4-methoxyglucobrassicin (4-methyoxy-3-indolylmethyl GSL)
ABA: abscisic acid
ACN: acetonitrile
ALY: glucoalyssin (5-methylsulphinylpentyl GSL)
ANOVA: analysis of variance
AtMYB: *Arabidopsis thaliana* MYB transcription factor
CO_2_: carbon dioxide
ERU: glucoerucin (4-methylthiobutyl GSL)
ESI: electron spray ionization
GBC: glucobrassicin (3-indolylmethyl GSL)
GSLs: glucosinolates
IBE: glucoiberin (3-methylsulfinylpropyl GSL)
JA: jasmonic acid
JA-Ile: 7-Jasmonoyl-L-isoleucine
LC-Q-TOF-MS: liquid chromatography-quadrupole time-of-flight mass spectrometry
MeOH: methanol
Met: methionine
NeoGB: neoglucobrassicin (1-methoxy-3-indolylmethyl GSL)
OPDA: 12-*oxo*-phytodienoic acid
PCA: principal component analysis
Phe: phenylalanine
RAPH: glucoraphanin (4-methylsulfinylbutyl GSL)
ROS: reactive oxygen species
SA: salicylic acid
Trp: tryptophan
UPLC: ultra-performance liquid chromatography

## References

[1] Rausher MD (2001). Co-evolution and plant resistance to natural enemies. Nature.

[2] Baldwin IT, Halitschke R, Kessler A, Schittko U (2001). Merging molecular and ecological approaches in plant-insect interactions. Curr Opin Plant Biol.

[3] Coley P (1988). Effects of plant growth rate and leaf lifetime on the amount and type of anti-herbivore defense. Oecologia.

[4] Bidart-Bouzat MG, Mithen R, Berenbaum MR (2005). Elevated CO_2_ influences herbivory-induced defense responses of *Arabidopsis thaliana*. Oecologia.

[5] Bekaert M, Edger PP, Hudson CM, Pires JC, Conant GC (2012). Metabolic and evolutionary costs of herbivory defense: systems biology of glucosinolate synthesis. New Phytol.

[6] Weaver AJ, Zickfeld K, Montenegro A, Eby M (2007). Long term climate implications of 2050 emission reduction targets. Geophys Res Lett.

[7] Edgerton MD (2009). Increasing crop productivity to meet global needs for feed, food, and fuel. Plant Physiol.

[8] Leakey ADB, Xu F, Gillespie KM, McGrath JM, Ainsworth EA, Ort DR (2009). Genomic basis for stimulated respiration by plants growing under elevated carbon dioxide. Proc Natl Acad Sci U S A.

[9] Schauer N, Fernie AR (2006). Plant metabolomics: towards biological function and mechanism. Trends Plant Sci.

[10] Graham N, May S, Schmidt R, Bancroft I (2011). Bioinformatics resources for *Arabidopsis thaliana*. Genetics and Genomics of the Brassicaceae.

[11] Mewis I, Appel HM, Hom A, Raina R, Schultz JC (2005). Major signaling pathways modulate Arabidopsis glucosinolate accumulation and response to both phloem-feeding and chewing insects. Plant Physiol.

[12] Winde I, Wittstock U (2011). Insect herbivore counteradaptations to the plant glucosinolate–myrosinase system. Phytochemistry.

[13] Reichelt M, Brown PD, Schneider B, Oldham NJ, Stauber E, Tokuhisa J, Kliebenstein DJ, Mitchell-Olds T, Gershenzon J (2002). Benzoic acid glucosinolate esters and other glucosinolates from *Arabidopsis thaliana*. Phytochemistry.

[14] Hirai MY (2009). A robust omics-based approach for the identification of glucosinolate biosynthetic genes. Phytochem Rev.

[15] Frerigmann H, Gigolashvili T (2014). MYB34, MYB51 and MYB122 distinctly regulate indolic glucosinolate biosynthesis in *Arabidopsis thaliana*. Mol Plant.

[16] Gigolashvili T, Berger B, Fluegge U-I (2009). Specific and coordinated control of indolic and aliphatic glucosinolate biosynthesis by R2R3-MYB transcription factors in *Arabidopsis thaliana*. Phytochem Rev.

[17] Gigolashvili T, Berger B, Mock H-P, Mueller C, Weisshaar B, Fluegge U-I (2007). The transcription factor HIG1/MYB51 regulates indolic glucosinolate biosynthesis in *Arabidopsis thaliana*. Plant J.

[18] Celenza JL, Quiel JA, Smolen GA, Merrikh H, Silvestro AR, Normanly J, Bender J (2005). The Arabidopsis ATR1 Myb transcription factor controls indolic glucosinolate homeostasis. Plant Physiol.

[19] Gigolashvili T, Yatusevich R, Berger B, Mueller C, Fluegge U-I (2007). The R2R3-MYB transcription factor HAG1/MYB28 is a regulator of methionine-derived glucosinolate biosynthesis in *Arabidopsis thaliana*. Plant J.

[20] Gigolashvili T, Engqvist M, Yatusevich R, Mueller C, Fluegge U-I (2008). HAG2/MYB76 and HAG3/MYB29 exert a specific and coordinated control on the regulation of aliphatic glucosinolate biosynthesis in *Arabidopsis thaliana*. New Phytol.

[21] Hirai MY, Sugiyama K, Sawada Y, Tohge T, Obayashi T, Suzuki A, Araki R, Sakurai N, Suzuki H, Aoki K, Goda H, Nishizawa OI, Shibata D, Saito K (2007). Omics-based identification of *Arabidopsis* Myb transcription factors regulating aliphatic glucosinolate biosynthesis. Proc Natl Acad Sci U S A.

[22] Sønderby IE, Geu-Flores F, Halkier BA (2010). Biosynthesis of glucosinolates - gene discovery and beyond. Trends Plant Sci.

[23] Beekwilder J, van Leewen W, van Dam NM, Bertossi M, Grandi V, Mizzi L, Soloviev M, Szabados L, Milthoff JW, Schipper B, Verbocht H, de Vos RC, Morandini P, Aarts MG, Bovy A (2008). The impact of the absence of aliphatic glucosinolates on insect herbivory in Arabidopsis. PLoS One.

[24] Sønderby IE, Burow M, Rowe HC, Kliebenstein DJ, Halkier BA (2010). A complex interplay of three R2R3 MYB transcription factors determines the profile of aliphatic glucosinolates in Arabidopsis. Plant Physiol.

[25] Karowe DN, Seimens DH, Mitchell-Olds T (1997). Species-specific response of glucosinolate content to elevated atmospheric CO_2_. J Chem Ecol.

[26] Schonhof I, Kläring H-P, Krumbein A, Schreiner M (2007). Interaction between atmospheric CO_2_ and glucosinolates in broccoli. J Chem Ecol.

[27] La GX, Fang P, Teng YB, Li YJ, Lin XY (2009). Effect of CO_2_ enrichment on the glucosinolate contents under different nitrogen levels in bolting stem of Chinese kale (*Brassica alboglabra* L.). J Zhejiang Univ Sci B.

[28] Klaiber J, Najar-Rodriguez AJ, Dialer E, Dorn S (2013). Elevated carbon dioxide impairs the performance of specialized parasitoid of an aphid host feeding on *Brassica* plants. Biol Control.

[29] Rosen C, Fritz V, Gardner G, Hecht S, Carmella S, Kenney P (2005). Cabbage yield and glucosinolate concentrations as affected by nitrogen and sulfur fertility. HortScience.

[30] Kim S-J, Matsuo T, Watanabe M, Watanabe Y (2002). Effect of nitrogen and sulphur application on the glucosinolate content in vegetable turnip rape (*Brassica rapa* L.). Soil Sci Plant Nutr.

[31] Aires A, Rosa E, Carvalho R (2006). Effect of nitrogen and sulfur fertilization on glucosinolates in the leaves and roots of broccoli sprouts (*Brassica oleracea* var. *italica*). J Sci Food Agric.

[32] Howe GA (2004). Jasmonates as signals in the wound response. J Plant Growth Reg.

[33] Mithöfer A, Boland W (2012). Plant defense against herbivores: chemical aspects. Annu Rev Plant Biol.

[34] Fonseca S, Chini A, Hamberg M, Adie B, Porzel A, Kramell R, Miersch O, Wasternack C, Solano R (2009). (+)-7-iso-Jasmonoyl-L-isoleucine is the endogenous bioactive jasmonate. Nat Chem Biol.

[35] Bonaventure G, Baldwin IT (2010). Transduction of wound and herbivory signals in plastids. Commun Integ Biol.

[36] Maruta T, Inoue T, Tamoi M, Yabuta Y, Yoshimura K, Ishikawa T, Shigeoka S (2011). *Arabidopsis* NADPH oxidases, AtrbohD and AtrbohF, are essential for jasmonic acid-induced expression of genes regulated by MYC2 transcription factor. Plant Sci.

[37] Erb M, Meldau S, Howe GA (2012). Role of phytohormones in insect-specific plant reactions. Trends Plant Sci.

[38] Jones JDG, Dangl JL (2006). The plant immune system. Nature.

[39] Pieterse CMJ, Van der Does D, Zamioudis C, Leon-Reyes A, Van Wees SCM (2012). Hormonal modulation of plant immunity. Annu Rev Cell Dev Biol.

[40] Robert-Seilaniantz A, Grant M, Jones JDG (2011). Hormone crosstalk in plant disease and defense: More than just JASMONATE-SALICYLATE antagonism. Annu Rev Phytopathol.

[41] Mikkelsen MD, Petersen BL, Glawischnig E, Jensen AB, Andreasson E, Halkier BA (2003). Modulation of CYP79 genes and glucosinolate profiles in Arabidopsis by defense signaling pathways. Plant Physiol.

[42] Schuster J, Knill T, Reichelt M, Gershenzon J, Binder S (2006). BRANCHED-CHAIN AMINOTRANSFERASE4 is part of the chain elongation pathway in the biosynthesis of methionine-derived glucosinolates in *Arabidopsis*. Plant Cell.

[43] Bodnaryk RP (1992). Effects of wounding on glucosinolates in the cotyledons of oilseed rape and mustard. Phytochemistry.

[44] Bidart-Bouzat MG, Imeh-Nathaniel A (2008). Global change effects on plant chemical defenses against insect herbivores. J Integr Plant Biol.

[45] Ainsworth EA, Rogers A (2007). The response of photosynthesis and stomatal conductance to rising CO_2_: mechanisms and environmental interactions. Plant Cell Environ.

[46] de Vos RC, Moco S, Lommen A, Keurentjes JJ, Bino RJ, Hall RD (2007). Untargeted large-scale plant metabolomics using liquid chromatography coupled to mass spectrometry. Nat Protoc.

[47] Reymond P, Bodenhausen N, Van Poecke RMP, Krishnamurthy V, Dicke M, Farmer EE (2004). A conserved transcript pattern in response to a specialist and a generalist herbivore. Plant Cell.

[48] Kos M, Houshyani B, Wietsma R, Kabouw P, Vet LEM, van Loon JJA, Dicke M (2012). Effects of glucosinolates on a generalist and specialist leaf-chewing herbivore and an associated parasitoid. Phytochemistry.

[49] Weigel D, Glazebrook J (2002). Arabidopsis, A Laboratory Manual.

[50] Marschner H (1995). Functions of mineral nutrients: macronutrients. Mineral Nutrition of Higher Plants.

[51] Loudet O, Chaillou S, Merigout P, Talbotec J, Daniel-Vedele F (2003). Quantitative trait loci analysis of nitrogen use in Arabidopsis. Plant Physiol.

[52] Boyes DC, Zayed AM, Ascenzi R, McCaskill AJ, Hoffman NE, Davis KR, Görlach J (2001). Growth stage–based phenotypic analysis of Arabidopsis: A model for high throughput functional genomics in plants. Plant Cell.

[53] Pluskal T, Castillo S, Villar-Briones A, Orešič M (2010). MZmine 2: modular framework for processing, visualizing, and analyzing mass spectrometry-based molecular profile data. BMC Bioinformatics.

[54] Xia J, Psychogios N, Young N, Wishart DS (2009). MetaboAnalyst: a web server for metabolomic data analysis and interpretation. Nucleic Acids Res.

[55] Xia J, Mandal R, Sinelnikov I, Broadhurst D, Wishart DS (2012). MetaboAnalyst 2.0 – a comprehensive server for metabolic data analysis. Nucleic Acids Res.

[56] Xia J, Sinelnikov I, Han B, Wishart, DS. MetaboAnalyst 3.0 – making metabolomics more meaningful. Nucleic Acids Research 2015. doi:10.1093/nar/gkv380. 10.1093/nar/gkv380 PMC4489235 25897128

[57] Pan X, Welti R, Wang X (2010). Quantitative analysis of major plant hormones in crude plant extracts by high-performance liquid chromatography-mass spectrometry. Nat Protoc.

[58] van Dam NM, Witjes L, Svantoš A (2004). Interactions between aboveground and belowground induction of glucosinolates in two wild *Brassica* species. New Phytol.

[59] Buchner R. Approach to determination of HPLC response factors for glucosinolates. In: Wathelet JP, editor. Glucosinolates in rapeseeds: Analytical aspects. Dordrecht; 1987. P. 50–58.

[60] EC. Oil seeds—determination of glucosinolates. High performance liquid chromatography. Communities. Official Journal of the European Communities L 170/28 Annex VIII, 27–34.1990.

[61] Brown PD, Tokuhisa JG, Reichelt M, Gershenzon J (2003). Variation of glucosinolate accumulation among different organs and developmental stages of *Arabidopsis thaliana*. Phytochemistry.

[62] Proietti S, Bertini L, Van der Ent S, Leon-Reyes A, Pieterse CMJ, Tucci M, Caporale C, Caruso C (2011). Cross activity of orthologous WRKY transcription factors in wheat and Arabidopsis. J Exp Bot.

[63] Zhao S, Fernald RD (2005). Comprehensive algorithm for quantitative real-time polymerase chain reaction. J Comput Biol.

[64] Sun Y, Guo H, Zhu-Salzman K, Ge F (2013). Elevated CO_2_ increases the abundance of the peach aphid on *Arabidopsis* by reducing jasmonic acid defenses. Plant Sci.

[65] Burow M, Rice M, Hause B, Gershenzon J, Wittstock U (2007). Cell- and tissue-specific localization and regulation of the epithiospecifier protein in *Arabidopsis thaliana*. Plant Mol Biol.

[66] Bloom AJ (2015). The increased importance of distinguishing among plant nitrogen sources. Curr Opin Plant Biol.

[67] Noctor G (2006). Metabolic signaling in defence and stress: the central roles of soluble redox couples. Plant Cell Environ.

[68] Paudel J, Copley T, Amirizian A, Prado A, Bede JC (2013). *Arabidopsis* redox status in response to caterpillar herbivory. Front Plant Sci.

