# Supplementary figures and images for: Effect of atmospheric carbon dioxide levels and nitrate fertilization on glucosinolate biosynthesis in mechanically damaged Arabidopsis plants
Source: BMC Plant Biol. 2016 Mar 22;16:68. doi: 10.1186/s12870-016-0752-1 (PMC4802917; doi:10.1186/s12870-016-0752-1)

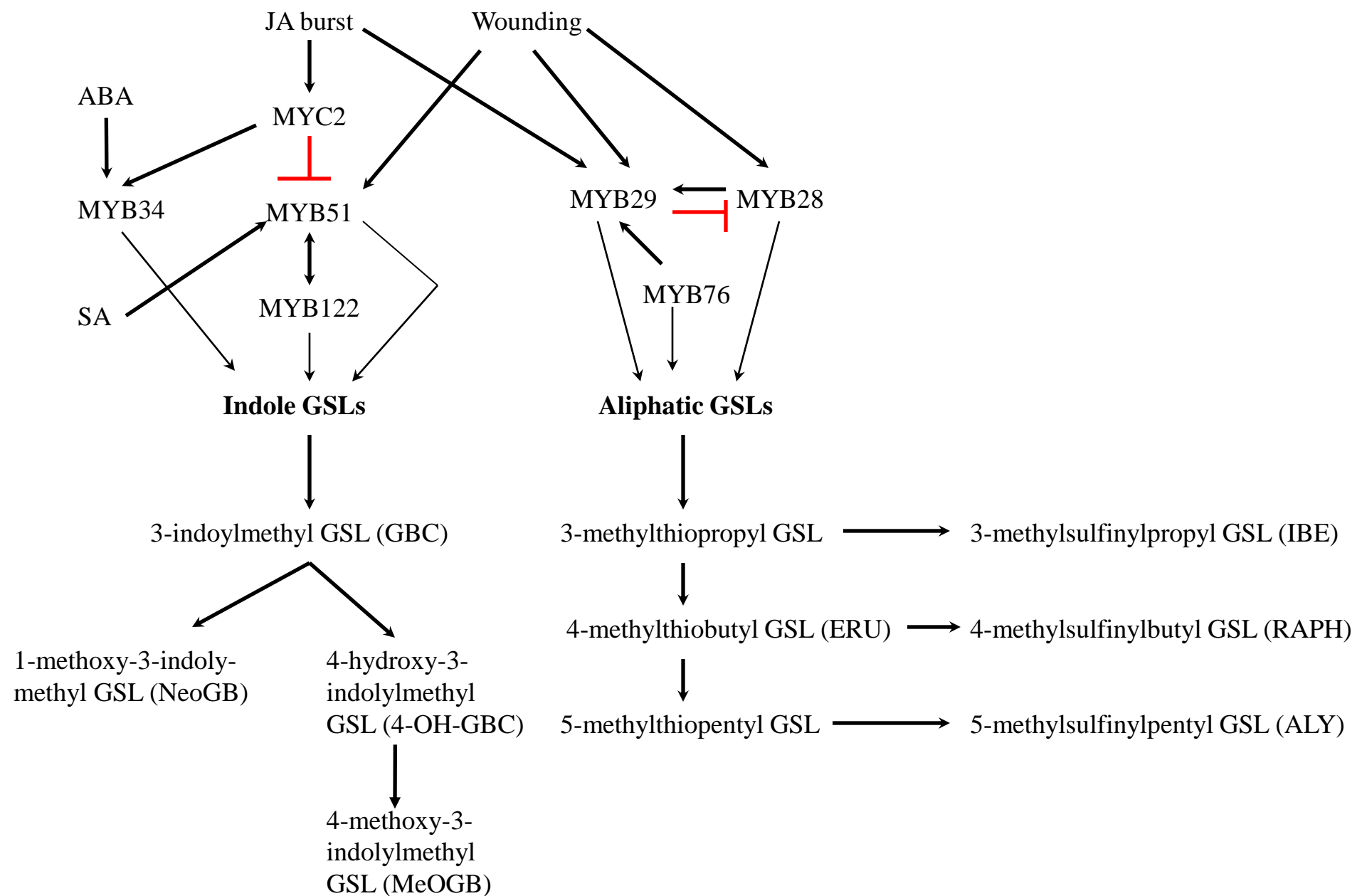

Supplement: Additional file 1: Figure S1. — MYB transcription factor regulation of glucosinolate biosynthesis in Arabidopsis. Wounding and wound-related stress hormones, such as jasmonic acid or methyl jasmonate, regulate MYB transcription factors that regulate glucosinolate biosynthesis. AtMYB28, AtMYB29 and AtMYB76 regulate expression of genes that encode enzymes in aliphatic GSL biosynthesis whereas AtMYB 34, AtMYB51 and AtMYB122 regulate expression of genes that encode enzymes in indole GSL biosynthesis. Abbreviations: abscisic acid: ABA, glucosinolate: GSL, jasmonic acid: JA, salicylic acid: SA. (PDF 7 kb) [file 12870_2016_752_MOESM1_ESM.pdf]
