# Supplementary material for: Effect of atmospheric carbon dioxide levels and nitrate fertilization on glucosinolate biosynthesis in mechanically damaged Arabidopsis plants
Source: BMC Plant Biol. 2016 Mar 22;16:68. doi: 10.1186/s12870-016-0752-1 (PMC4802917; doi:10.1186/s12870-016-0752-1)
Supplement: Additional file 3: Table S2. — Primers used for quantitative real time-polymerase chain reaction (qRT-PCR) (DOC 40 kb) [file 12870_2016_752_MOESM3_ESM.doc]

Supplemental Table 2. Primers for quantitative real time-polymerase chain reaction (qRT-PCR).

| Gene | Annealing temperature (°C) | Forward (5’-3’) | Reverse (5’-3) | Ref. |
| --- | --- | --- | --- | --- |
| Detection of genomic contamination: | | | | |
| AtEIL2 |  | CAGATTCTATGGATATGTATAACAACAA | GTAAAGAGCAGCGAGCCATAAAG | [1] |
| Reference Genes: | | | | |
| AtACT2  (At5g09810) | 60 | GTATGCTCTTCCTCATGCTATCCTT | TTCCCGTTCTGCGGTAGTG | [2] |
| AtUnk  (At4g26410) | 60 | GAGCTGAAGTGGCTT CCATGAC | GGTCCGACATACCCATGATCC | [3] |
| AtUBC  (At5g25760) | 58 | GCAGTTGACAATTCG TTCTCT | GAGCGGTCCATTTGAATATGTT | Primer3 |
| *Genes-of-interest:* | | | | |
| AtMYB28 | 57 | TCTGATTAGGGTTGAAACGGTGTGG | CGACCACTTGTTGCCACGAGA | Primer3 |
| MYB29 | 58 | GGCAACAAGTGGTCAGTCATAGCG | TTGAGTCATAGGCAAGTGGCTTGTG | Primer3 |
| MYB76 | 57 | TCGTGGCAATAAGTGGTCTGTCATA | GGGTTAGAAGAAGCTAGTGGCTTGT | Primer3 |
| MYB34 | 57 | TAAGGGTAACAAGTGGGCCGC | GATGCCTTTTTGCTTCAACCGCT | Primer3 |
| MYB51 | 57 | TCACGGCAACAAATGGTCTGCT | CGGTACCGGAGGTTATGCCC | Primer3 |
| MYB122 | 58 | CATGGCAACAAATGGTCGGCC | CCGGCTCCATCGAGAAGGGAT | Primer3 |

[1] Proietti S, Bertini L, Van der Ent S, Leon-Reyes A, Pieterse CMJ, Tucci M, Caporale C, Caruso C. Cross activity of orthologous WRKY transcription factors in wheat and *Arabidopsis*. Journal of Experimental Botany 2011;62:1975-1990.

[2] Beste L, Nahar N, Dalman K, Fujioka S, Jonsson L, Dutta PC, Sitbon F. Synthesis of hydroxylated sterols in transgenic *Arabidopsis* plants alters growth and steroid metabolism. Plant Physiology 2011;157:426-440.

[3] Czechowski T, Stitt M, Altmann T, Udvardi MK, Scheible W-R. Genome-wide identification and testing of superior reference genes for transcript normalization in Arabidopsis. Plant Physiology 2005;139:5-17.
