# Supplementary material for: Effect of atmospheric carbon dioxide levels and nitrate fertilization on glucosinolate biosynthesis in mechanically damaged Arabidopsis plants
Source: BMC Plant Biol. 2016 Mar 22;16:68. doi: 10.1186/s12870-016-0752-1 (PMC4802917; doi:10.1186/s12870-016-0752-1)
Supplement: Additional file 4: Table S3. — Statistical analysis of phytohormone, AtAMB gene expression and glucosinolate levels. (DOC 94 kb) [file 12870_2016_752_MOESM4_ESM.doc]

**Supplemental Table 3. Statistical analyses of A) phytohormones B) *AtMYB* gene expression and C) glucosinolates.** Significant differences in gene expression and metabolite levels were analyzed by 3-factorial ANOVA. When a significant interaction was detected, it was followed by a 2-way ANOVA between treatments.

| 1. **Phytohormones** | | | |
| --- | --- | --- | --- |
| **Hormones** | **Interaction/ Treatment**  **effect** |  | |
| **JA** | C x N x W | F_(1, 21)_ = 5.38, p = 0.03 | |
|  |  | **Ambient CO_2_(440 ppm)** | **Elevated CO_2_(880 ppm)** |
|  | N x W | F_(1,11)_ = 0.99, p = 0.34 | F_(1,11)_ = 6.64, p = 0.03 |
| **JA-Ile** | C x N x W | F_(1, 20)_ = 18.7, p < 0.01 | |
|  |  | **Ambient CO_2_(440 ppm)** | **Elevated CO_2_(880 ppm)** |
|  | N x W | F_(1, 10)_ = 1.50, p = 0.25 | F_(1, 10)_ = 26.73, p <0.01 |
| **OPDA** | N x W | F_(1, 24)_ = 4.40, p = 0.047 | |
|  |  | **1 mM N** | **10 mM N** |
|  | W | F_(1,12)_ = 18.04, p = 0.001 | F_(1,12)_ = 0.08, p = 0.79 |
| **SA** | C x N x W | F_(1, 24)_ = 0.27, p = 0.61 | |
| **ABA** | C x N x W | F_(1, 23)_ = 2.99, p = 0.10 | |

| **B)** ***AtMYB* gene expression** | | | |
| --- | --- | --- | --- |
| **Genes** | **Interaction/ Treatment effect** |  | |
| ***AtMYB28*** | C x N | F_(1,24)_ = 4.28, p = 0.049 | |
|  |  | **Ambient CO_2_(440 ppm)** | **Elevated CO_2_(880 ppm)** |
|  | N | F_(1,12)_ = 0.65, p = 0.44 | F_(1,12)_ = 3.69, p = 0.079 |
|  | W | F_(1,12)_ = 11.27, p = 0.006 | F_(1,12)_ = 1.31, p = 0.275 |
| ***AtMYB29*** | N | F_(1,24)_ = 5.22, p = 0.032 | |
|  | W | F_(1,24)_ = 14.94, p = 0.001 | |
| ***AtMYB76*** | C x N | F_(1,24)_ = 6.23 p = 0.020 | |
|  |  | **Ambient CO_2_(440 ppm)** | **Elevated CO_2_(880 ppm)** |
|  | N | F_(1,12)_ = 1.89, p = 0.21 | F_(1,12)_ = 4.15, p = 0.06 |
|  | W | F_(1,24)_ = 6.39, p = 0.019 | |
| ***AtMYB34*** | C | F_(1,24)_ = 11.80, p = 0.002 | |
|  | W | F_(1,24)_ = 5.93, p = 0.023; however, these differences were not detectable by t-test | |
| ***AtMYB51*** | C | F_(1,24)_ = 10.95, p = 0.003 | |

| 1. **Glucosinolates** | | | | |
| --- | --- | --- | --- | --- |
| **Glucosinolates (GSL)** | **Interaction/ Treatment**  **effect** |  | | |
| **Total GSL** | C x N x W | F_(1, 24)_ = 5.59, p = 0.026 | | |
|  |  | **Ambient CO_2_ (440 ppm)** | **Elevated CO_2_ (880 ppm)** | |
|  | N x W | F_(1,12)_ = 0.22, p = 0.65 | F_(1,12)_ = 9.2, p = 0.011 | |
|  | N | F_(1, 24)_ = 4.25, p = 0.05 | | |
|  | W | F_(1,24)_ = 8.23, p = 0.008 | | |
| **Indole GSL** | C x N x W | F_(1, 24)_ = 4.26, p = 0.05 | | |
|  |  | **Ambient CO_2_ (440 ppm)** | | **Elevated CO_2_ (880 ppm)** |
|  | N x W | F_(1,12)_ = 0.06, p = 0.82 | | F_(1,12)_ = 7.4, p = 0.019 |
|  | W | F_(1, 24)_ = 46.77, p < 0.001 | | |
| **Aliphatic GSL** | C x N x W | F_(1, 24)_ = 5.11, p = 0.033 | | |
|  |  | **Ambient CO_2_ (440 ppm)** | **Elevated CO_2_ (880 ppm)** | |
|  | N x W | F_(1,12)_ = 0.24, p = 0.24 | F_(1,12)_ = 8.45, p = 0.013 | |
|  |  |  | | |
| **Indole GSLs** | **Interaction/ Treatment**  **effect** |  | | |
| **Glucobrassicin (GBC)**  3-indolylmethyl GSL | C | F_(1, 24)_ = 4.86, p = 0.037 | | |
|  | W | F_(1, 24)_ = 36.10, p < 0.001 | | |
| **Methoxygluco-brassicin (4MeOGB)**  4-methoxy-3-indolylmethyl GSL | C | F_(1, 24)_ = 18.71, p < 0.001 | | |
| **Neo-glucobrassicin (NeoGB)**  1-methyoxy-3-indolylmethyl GSL | N x W | F_(1, 24)_ = 24.01, p < 0.001 | | |
|  |  | **1 mM N** | | **10 mM N** |
|  | W | F_(1, 12)_ = 43.34, p < 0.001 | | F_(1, 12)_ = 0.30, p = 0.60 |
|  | C | F_(1, 24)_ = 18.02, p < 0.001 | | |
|  |  |  |  |  |
|  |  |  | | |
| **Aliphatic GSLs** | **Interaction/ Treatment**  **effect** |  | | |
| **Glucoiberin (IBE)**  3-methylsulfinylpropyl GSL | C x N | F_(1, 24)_ = 5.17, p = 0.032 | | |
|  |  | **Ambient CO_2_ (440 ppm)** | | **Elevated CO_2_ (880 ppm)** |
|  | N | F_(1,12)_ = 6.32, p = 0.03 | | F_(1,12)_ = 0.53, p = 0.48 |
| **Glucoerucin**  **(ERU)**  4-methylthiobutyl GSL | C x N | F_(1, 24)_ = 9.91, p = 0.004 | | |
|  |  | **Ambient CO_2_ (440 ppm)** | | **Elevated CO_2_ (880 ppm)** |
|  | N | F_(1,12)_ = 2.35, p = 0.15 | | F_(1,12)_ = 11.35, p = 0.006 |
|  | C | F_(1, 24)_ = 4.49, p = 0.45 | | |
| **Glucoraphanin (RAPH)**  4-methylsulfinylbutyl GSL | C x N x W | F_(1, 24)_ = 5.72, p = 0.025 | | |
|  |  | **Ambient CO_2_ (440 ppm)** | | **Elevated CO_2_ (880 ppm)** |
|  | N x W | F_(1, 12)_ = 0.26, p = 0.62 | | F_(1, 12)_ = 9.93, p = 0.008 |
|  | N | F_(1, 24)_ = 4.74, p = 0.04 | | |
| **Glucoalyssin (Aly)**  5-methylsulphinylpentyl | W | F_(1, 24)_ = 5.38, p = 0.029 | | |
|  | N | F_(1, 24)_ = 4.73, p = 0.04 | | |
