# Supplementary material for: Effect of atmospheric carbon dioxide levels and nitrate fertilization on glucosinolate biosynthesis in mechanically damaged Arabidopsis plants
Source: BMC Plant Biol. 2016 Mar 22;16:68. doi: 10.1186/s12870-016-0752-1 (PMC4802917; doi:10.1186/s12870-016-0752-1)

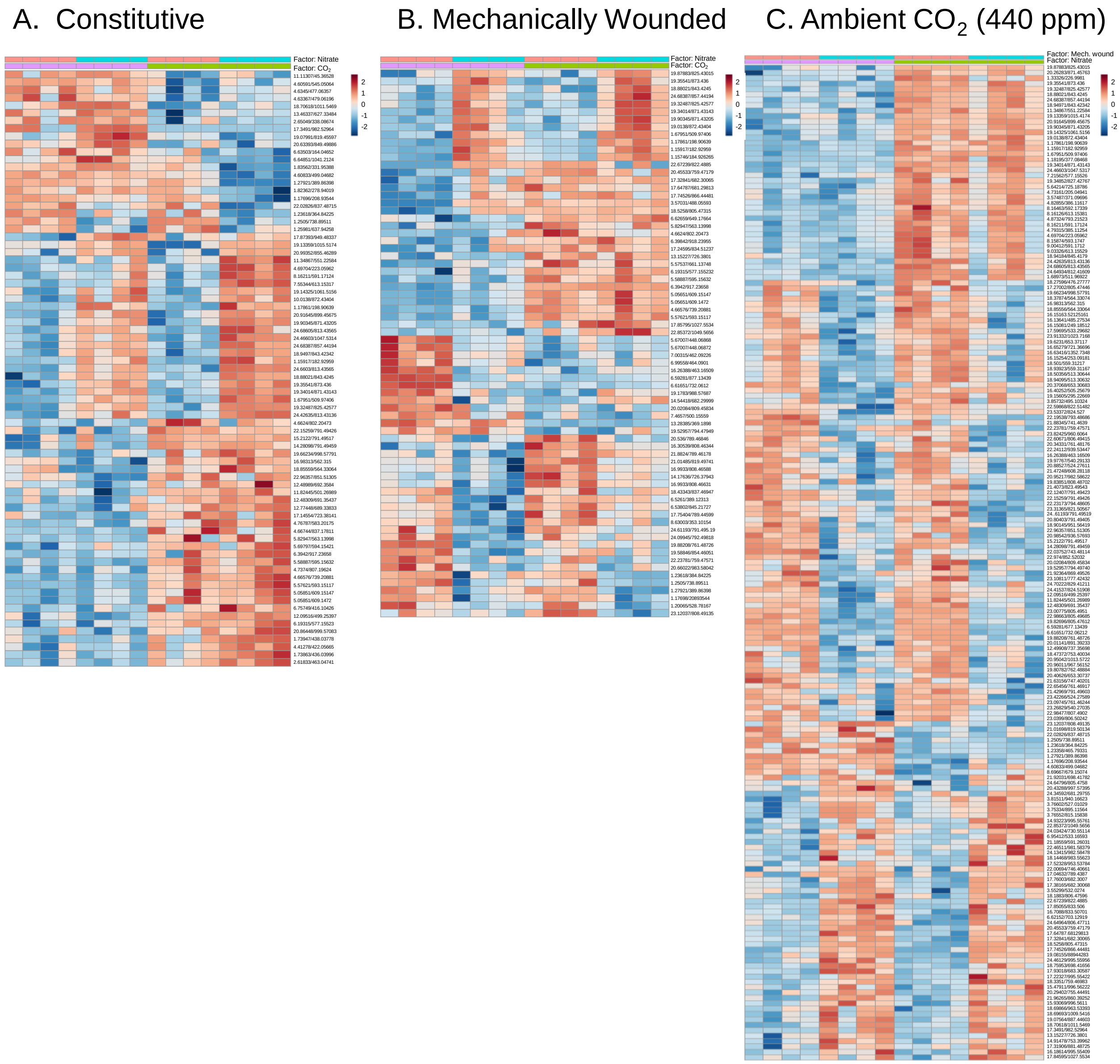
A. Constitutive
B. Mechanically Wounded
C. Ambient CO2 (440 ppm)
Factor: Nitrate
Factor: CO2
Factor: Mech. wound
2
1
0
-1
-2

D. Elevated CO2 (880 ppm)

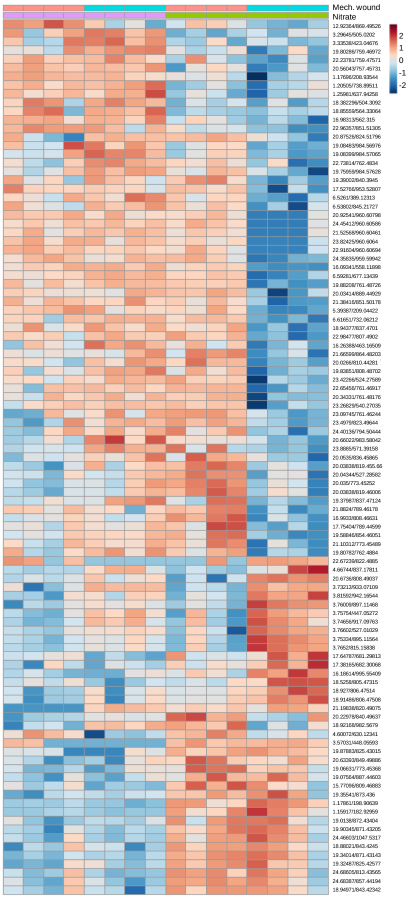

E. 1 mM Nitrate

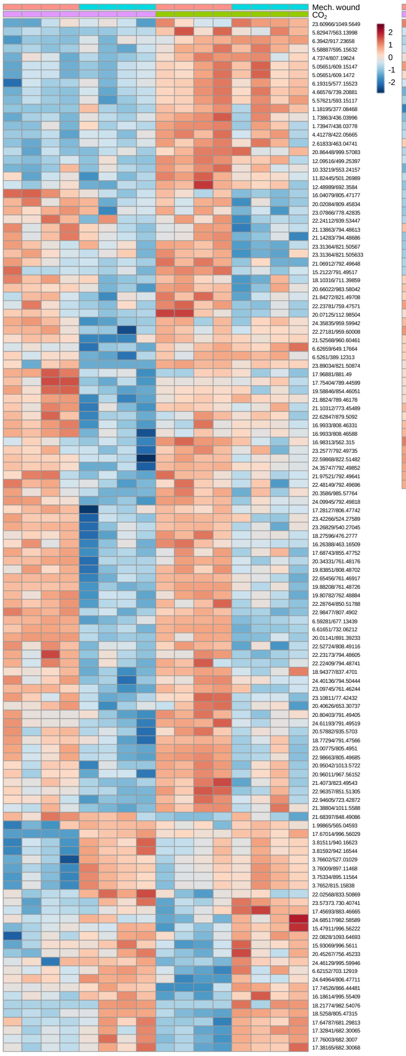

F. 10 mM Nitrate

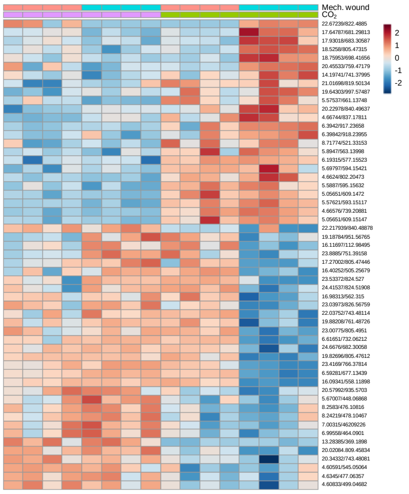

Supplement: Additional file 5: Figure S2. — Heat map of foliar metabolite profiles of Arabidopsis grown at different CO2levels, nitrate fertilization and wounding stress. Plants were grown under two different atmospheric CO2 levels (ambient (440 ppm; LC) or elevated (880 ppm; HC)) and fertilized with either 1 mM (LN) or 10 mM (HN) nitrate and either not treated (control) or mechanically damaged (wound). The average of 4 independent samples were compared by 2-way analysis of variance. Metabolites are identified by retention time/mass over charge ratio (RT/m/z). A) Constitutive foliar metabolic profile. Factors; 1 mM nitrate (blue), 10 mM nitrate (pink), 440 ppm CO2 (green) and 880 ppm CO2 (purple). B) Wound-induced metabolite profile. Factors; 1 mM nitrate (blue), 10 mM nitrate (pink), 440 ppm CO2 (green) and 880 ppm CO2 (purple). C) Metabolite profile of plants grown at ambient CO2 levels (440 ppm). Factors; constitutive (pink), mechanically damaged (blue), 1 mM nitrate (green) and 10 mM nitrate (purple). D) Metabolite profile of plants grown at elevated CO2 levels (880 ppm). Factors; constitutive (pink), mechanically damaged (blue), 1 mM nitrate (green) and 10 mM nitrate (purple). E) Metabolite profile of plants fertilized with 1 mM nitrate. Factors; control (pink), mechanically damaged (blue),440 ppm CO2 (green) and 880 ppm CO2 (purple). F) Metabolite profile of plants fertilized with 10 mM nitrate. Factors; control (pink), mechanically damaged (blue),440 ppm CO2 (green) and 880 ppm CO2 (purple). (PDF 204 kb) [file 12870_2016_752_MOESM5_ESM.pdf]
